# La importancia de la comunicación en salud

**DOI:** 10.7705/biomedica.8102

**Published:** 2025-08-11

**Authors:** Enrique Ardila, Carlos Arturo Hernández

**Affiliations:** 1 Comité Editorial, revista Biomédica revista Biomédica revista Biomédica Colombia

Ileana María Petra Micu, coordinadora de Enseñanza del Departamento de Psiquiatría y Salud Mental de la Universidad Nacional Autónoma de México, afirma:

*“A la comunicación se le considera como un proceso de comprensión y de compartir el significado de algo, es el elemento básico de la interacción humana que permite a las personas establecer, mantener y mejorar el contacto con otros, así como participar en el conocimiento de uno mismo. Simultáneamente es vista como una habilidad, y al mismo tiempo una forma de mostrar la personalidad dentro de una relación”*^1^.

Se reconoce la existencia de varios tipos de comunicación entre personas, a saber: la oral o verbal, es decir, lo que se dice; la paraverbal, la forma como se dice y que incluye el tono y el volumen de la voz, y la no verbal, es decir, los gestos, las posturas, el contacto visual, etc. Estas expresiones se deben integrar de forma coherente para que transmitan mensajes efectivos y eviten ambigüedades en su interpretación. Por ejemplo, una explicación empática puede verse invalidada si el tono utilizado es distante o si se evita el contacto visual con el receptor del mensaje. No sobra mencionar que, al igual que sucede en cualquier diálogo, la comunicación incluye no solo el aspecto lingüístico sino también el psicológico.

En medicina, tal vez la comunicación más básica, es aquella relacionada con la atención y cuidado de los pacientes y que podríamos llamar *comunicación clínica*. Se establece primordialmente en la relación médico-paciente, y se hace con el ánimo de elaborar una anamnesis lo más completa posible y formular posibles vías de resolver el motivo de la consulta.

Existen diferentes métodos de acercamiento al paciente según la situación por la que está atravesando, su estado de salud en general y el sitio en donde se encuentra. El paciente puede estar solo o acompañado de familiares o amigos; puede encontrarse en su casa -en su medio y entorno-, o asistir a una cita por consulta externa en un centro de salud o en un hospital, o encontrarse hospitalizado en salas comunes o en unidades especializadas como la de cuidados intensivos.

Al establecerse una buena comunicación con el paciente, se disminuye la angustia que pueda estar sintiendo y se evita su posible depresión, lográndose obtener una mejor historia clínica y, posiblemente, mejores alternativas para la resolución de sus síntomas y mejor aceptación del tratamiento propuesto. Por otro lado, el profesional médico va a estar menos expuesto a tener que enfrentar problemas legales por parte del paciente o de su familia, debido a falta de información clara y precisa sobre su enfermedad o tratamiento.

En algunos estudios se ha observado que la comunicación con el paciente es diferente según el sexo del médico: las mujeres se preocupan más por ciertos detalles como los sentimientos, mientras que los hombres están más centrados en resolver los problemas ^2^. Asimismo, la forma como se transmite el mensaje puede ayudar o dificultar el proceso de comunicación, especialmente cuando se intercambian ideas con personas que pertenecen a otras culturas, o que tienen un nivel de formación o un idioma diferentes y que, por supuesto, se expresan de otra manera ^3^.

Valga la pena llamar la atención a que, con el advenimiento de las nuevas tecnologías en medicina, se ha ido perdiendo la costumbre de elaborar una buena historia clínica y, es más, en muchas facultades de medicina le vienen dando cada vez menos importancia a este componente del ejercicio clínico.

Acá, sin lugar a duda, también se puede ubicar la comunicación con los familiares del paciente o sus amigos pues, obviamente, los miembros de su entorno familiar y social pueden estar igualmente deseosos de comprender mejor la alteración de su salud, su posible tratamiento y el pronóstico. No obstante, esto no lo piensa el médico tratante sino hasta que tiene que hacerlo y cae en cuenta de que en ningún momento se nos impartieron instrucciones sobre cómo informar las malas noticias al paciente o a sus familiares.

Un segundo nivel de comunicación es la que se da en las instituciones de salud como parte del cuidado del paciente y la labor docente formativa. La podríamos denominar *comunicación entre pares*, bien sea con enfermeras y ayudantes de enfermería y laboratorio, y que se da principalmente mediante las instrucciones que se consignan por escrito en la historia clínica del paciente. Asimismo, debe haber un intercambio de opiniones entre colegas en reuniones del servicio, en juntas médicas o en reuniones más elaboradas, como clases, conferencias, foros, congresos, etc.

Existe un tercer nivel de comunicación igualmente importante, el cual es la comunicación de los hallazgos de las diversas investigaciones en cualquier aspecto de la medicina; en otras palabras, la difusión del *conocimiento científico* obtenido para todo aquel que pueda estar interesado en el tema.

Es posible que las comunicaciones deban orientarse al campo de la salud pública dado que, eventualmente, pueden influir en la formulación de políticas oficiales. Este tipo de comunicación puede ser oral o escrita. La primera está dirigida principalmente a los funcionarios tomadores de decisiones, es decir, directivas sanitarias y sus equipos de salud, periodistas, dirigentes universitarios, líderes y el público en general. La segunda son los informes sobre brotes, epidemias, recomendaciones para la comunidad, estrategias en salud pública, entre otros muchos más, y que en la actualidad se divulgan en internet -redes sociales, chats, blogs- y en los medios tradicionales de la prensa escrita, la radio y la televisión.

Finalmente, debemos considerar a las publicaciones médicas. Este tipo de comunicación implica aprender a leer y analizar los datos, los cuales sirven, en muchas ocasiones, de punto de partida para adelantar una investigación, elaborar el protocolo de desarrollo y culminar este proceso en la publicación de un artículo -ojalá en una revista de nuestro hemisferio para que lo comunicado pueda causar mayor impacto.

A lo largo de su evolución como campo de estudio, desde el siglo XIX hasta nuestros días, la comunicación de la ciencia o del conocimiento científico ha recibido distintos nombres: popularización, vulgarización, diseminación, comprensión pública, apropiación social y educación científica, entre otros ^4^.

En Colombia, el término *apropiación* ha sido el designado para agrupar los esfuerzos, iniciativas y proyectos alrededor de la comunicación científica. Desde el 2005, se convirtió en política pública con la publicación de un documento elaborado por el entonces Departamento Administrativo de Ciencia, Tecnología e Innovación, Colciencias; a partir de este, se creó el Ministerio de Ciencia, Tecnología e Innovación mediante la Ley 1951 de 2019, cuyo propósito es impulsar la promoción del conocimiento y la productividad, y contribuir al desarrollo y la competitividad del país, así como construir una sociedad más equitativa ^5^.

Además, tiene importantes funciones, como evaluar y analizar las diferentes publicaciones realizadas en el país y, junto con las universidades públicas o privadas, contribuir en la formación de investigadores que ayuden en la generación de conocimiento útil en el desarrollo colombiano.

En suma, la comunicación en salud ya sea en la relación médico-paciente, en el trabajo con diferentes comunidades o en la difusión científica, es una competencia transversal que debe fortalecerse en todos los niveles de formación y ejercicio profesional. Es esencial que las facultades de medicina entrenen a los estudiantes en su manejo. Más allá de ser una herramienta técnica, la comunicación constituye un elemento fundamental de la atención en salud, la ética médica y la construcción de confianza. El reforzarla implica formar mejores profesionales, mejorar la calidad del servicio, y contribuir a una sociedad más informada, participativa y saludable.
